# Pretreatment CT radiomics for prediction of pathological complete response after neoadjuvant chemoimmunotherapy in NSCLC: a multicenter study incorporating habitat analysis

**DOI:** 10.3389/fimmu.2026.1933861

**Published:** 2026-09-23

**Authors:** Sunyi Zheng, Shuyuan Huang, Jiahui Zhang, Xiaomeng Yang, PengChun Ye, Weiping Wang, Wenhua Li, Houpu Liu, Chunlin Zhang, Guoqing Liao, Haiyu Zhou, Jing Wang, Xiaonan Cui, Dongsheng Yue

**Affiliations:** 1 Department of Radiology, Medical Artificial General Intelligence for Computation (MAGIC) Lab, National Clinical Research Center for Cancer, Tianjin’s Clinical Research Center for Cancer, State Key Laboratory of Druggability Evaluation and Systematic Translational Medicine, Tianjin Key Laboratory of Digestive Cancer, Tianjin Key Laboratory of Cancer Prevention and Therapy, Tianjin Medical University Cancer Institute and Hospital, Tianjin, China; 2 Department of Lung Cancer, Key Laboratory of Cancer Prevention and Therapy, National Clinical Research Center of Cancer, Lung Cancer Center, Tianjin Medical University Cancer Institute and Hospital, Tianjin, China; 3 Academy of Medical Engineering and Translational Medicine, Tianjin University, Tianjin, China; 4 National Clinical Research Center for Cancer, Tianjin’s Clinical Research Center for Cancer, State Key Laboratory of Druggability Evaluation and Systematic Translational Medicine, Tianjin Key Laboratory of Cancer Prevention and Therapy, Tianjin Medical University Cancer Institute and Hospital, Tianjin, China; 5 Department of Radiology, Ordos Central Hospital, Ordos, China; 6 Department of Thoracic Surgery, Guangdong Provincial People’s Hospital, Guangdong Academy of Medical Sciences, Southern Medical University, Guangzhou, China; 7 School of Public Health, Tianjin University of Traditional Chinese Medicine, Tianjin, China

**Keywords:** habitat, neoadjuvant chemoimmunotherapy, non-small cell lung cancer, pathologic complete response, radiomics

## Abstract

**Background:**

Pathological complete response (pCR) after neoadjuvant chemoimmunotherapy (NACI) varies among patients with non-small cell lung cancer (NSCLC). This study aimed to quantify intratumoral heterogeneity using habitat analysis and to develop a pretreatment model for predicting pCR.

**Methods:**

In this retrospective multicenter study, 287 patients with NSCLC who received NACI followed by surgery were included. Patients were randomly divided into a training cohort (n = 147) and an internal validation cohort (n = 63), with an independent external test cohort (n = 77). K-means clustering was used to partition tumors into three habitat subregions based on entropy. Radiomics features were extracted from pretreatment CT images at the whole-tumor and habitat levels. Whole-tumor, habitat-based, and combined models were developed to predict pCR. Model performance was evaluated using area under the curve (AUC) analysis, and model interpretability was assessed using Shapley Additive exPlanations (SHAP).

**Results:**

The habitat-based model showed acceptable predictive performance across the training, internal validation, and external test cohorts, with AUCs of 0.74, 0.71, and 0.67, respectively, and showed higher AUCs compared with the whole-tumor radiomics model, which achieved AUCs of 0.72, 0.68, and 0.65. The combined model achieved performance comparable to the habitat-based model. SHAP analysis suggested that, among the habitat-derived features, the most influential predictive features were primarily associated with the high-entropy habitat subregion.

**Conclusion:**

The habitat-based model shows potential performance for predicting pCR, and features from a high-entropy habitat subregion may provide complementary imaging information for preoperative assessment of treatment response.

## Introduction

Lung cancer is the most common type of cancer and the leading cause of cancer deaths worldwide. Among these, non-small cell lung cancer (NSCLC) is the predominant pathological subtype, accounting for approximately 85% of all lung cancers, and is associated with a 5-year survival rate of only 10% to 20% (1). Neoadjuvant chemoimmunotherapy (NACI) is an emerging preoperative approach for resectable NSCLC, which aims to shrink tumors and reduce disease stage before surgery, thereby lowering the risk of postoperative recurrence and extending patient survival (2). In recent years, several clinical trials have shown that NACI improves major pathological response (MPR) rates, with a subset of patients achieving pathological complete response (pCR) (3–6). Notably, pCR has been associated with favorable long-term outcomes in patients with resectable NSCLC (7–9). Despite these encouraging results, not all patients derive benefit from this treatment strategy.

To predict pathological response and guide patient selection for NACI, a range of biomarkers has been explored. Previous studies have reported that invasive markers, including programmed death-ligand 1 (PD-L1) expression, tumor mutational burden (TMB), and tumor-infiltrating lymphocytes (TILs), are associated with treatment response (10, 11). However, these approaches rely primarily on biopsy-based assessments, which are inherently limited by restricted sampling and therefore fail to fully capture intratumoral heterogeneity. Blood-based inflammatory markers, such as the neutrophil-to-lymphocyte ratio (NLR) (12) and the systemic immune inflammation index (SII) (13), have also been found to correlate with pCR following NACI. Their clinical applicability, however, is limited by the lack of standardized and universally accepted thresholds for reliable response prediction. For radiologic assessment of treatment response, the Response Evaluation Criteria in Solid Tumors (RECIST) based on CT are widely used. Nevertheless, RECIST relies exclusively on changes in maximal tumor diameter, shows poor correlation with pCR after NACI, and fails to reliably distinguish delayed response or immunotherapy-related pseudoprogression. Consequently, treatment efficacy cannot be accurately assessed using conventional size-based imaging criteria alone (14, 15). These limitations highlight the need for a reliable, noninvasive approach that enables early assessment of NACI efficacy, avoids ineffective interventions, and supports optimized treatment decision-making.

Radiomics is a noninvasive quantitative imaging approach that extracts high throughput, multidimensional features from CT images to characterize intratumoral heterogeneity across the entire tumor volume (16, 17). It has been increasingly explored in lung cancer for applications including early diagnosis, treatment response assessment, and prognostic evaluation (18–21). Studies have found that both tumoral and peritumoral radiomic features are associated with response to immunotherapy (22, 23). However, most radiomics studies consider the tumor as a single homogeneous entity, which may limit their ability to capture its intrinsic spatial heterogeneity. During tumor progression, variations in the local microenvironment, such as vascular distribution, cellular density, and immune infiltration, occur across different regions of the tumor, leading to heterogeneous biological phenotypes and variable therapeutic sensitivity (24).

Habitat analysis seeks to address this limitation by partitioning tumors into multiple intratumoral subregions, each reflecting distinct microstructural and biological characteristics. This subregional characterization allows for a more detailed representation of local spatial heterogeneity and facilitates the assessment of structural and functional differences among tumor habitats, thereby providing additional insight into tumor behavior and treatment response (25). To date, the role of CT-based habitat radiomics in pretreatment prediction of pathological response to NACI has not been fully explored, and its clinical relevance remains to be established.

Therefore, this study aims to develop and validate a habitat-based radiomics model on pretreatment CT scans for predicting pCR following NACI in patients with NSCLC, thereby providing a basis for exploratory pretreatment risk stratification.

## Materials and methods

### Study population

This multicenter retrospective study was conducted in accordance with the Declaration of Helsinki and was approved by the ethics committees of Tianjin Medical University Cancer Institute and Hospital (BC20252896) and Guangdong Provincial People’s Hospital (KY202509302). Due to its retrospective design, the requirement for patient informed consent was waived. This study retrospectively collected 295 patients with NSCLC who received NACI and subsequently underwent surgery, including 212 patients from Tianjin Medical University Cancer Institute and Hospital (TMUCIH) between August 2018 and November 2022 and 83 patients from Guangdong Provincial People’s Hospital (GPPH) between September 2019 and March 2023. All patients underwent non-contrast CT scans prior to NACI to assess tumor status and lymph node involvement. The inclusion criteria were as follows: (1) histopathological examination confirmed the diagnosis of clinical stage II–III NSCLC; (2) CT-detectable tumors were present, and image quality was sufficient for radiologic evaluation; (3) neoadjuvant immunotherapy combined with platinum-based chemotherapy was administered prior to surgical intervention. The exclusion criteria were as follows: (1) absence of pretreatment CT images or inadequate image quality that precluded radiologic analysis; (2) unclear tumor boundaries that could not be reliably delineated; (3) incomplete clinical or pathological data; (4) clinical stage I or IV disease before treatment; (5) receipt of any prior lung cancer treatment before neoadjuvant therapy. Patients from TMUCIH were stratified by pCR status and randomly allocated to a training cohort and an internal validation cohort at a 7:3 ratio to preserve the outcome distribution, whereas patients from GPPH served as an independent external test cohort. The patient selection flowchart is presented in **Figure 1**, and an overview of the study workflow is provided in **Figure 2**.

**Figure 1:**
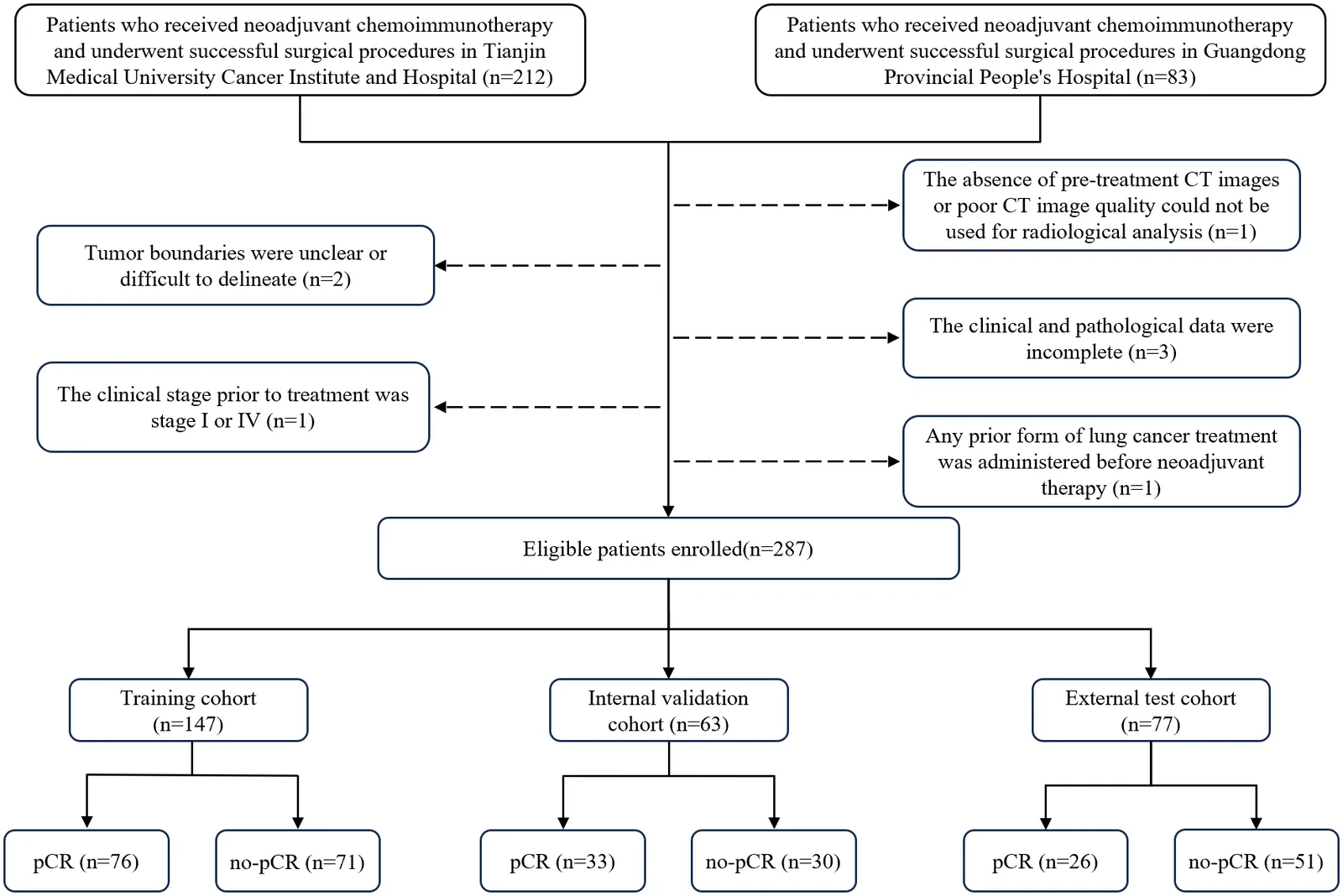
Flowchart of patient selection.

**Figure 2:**
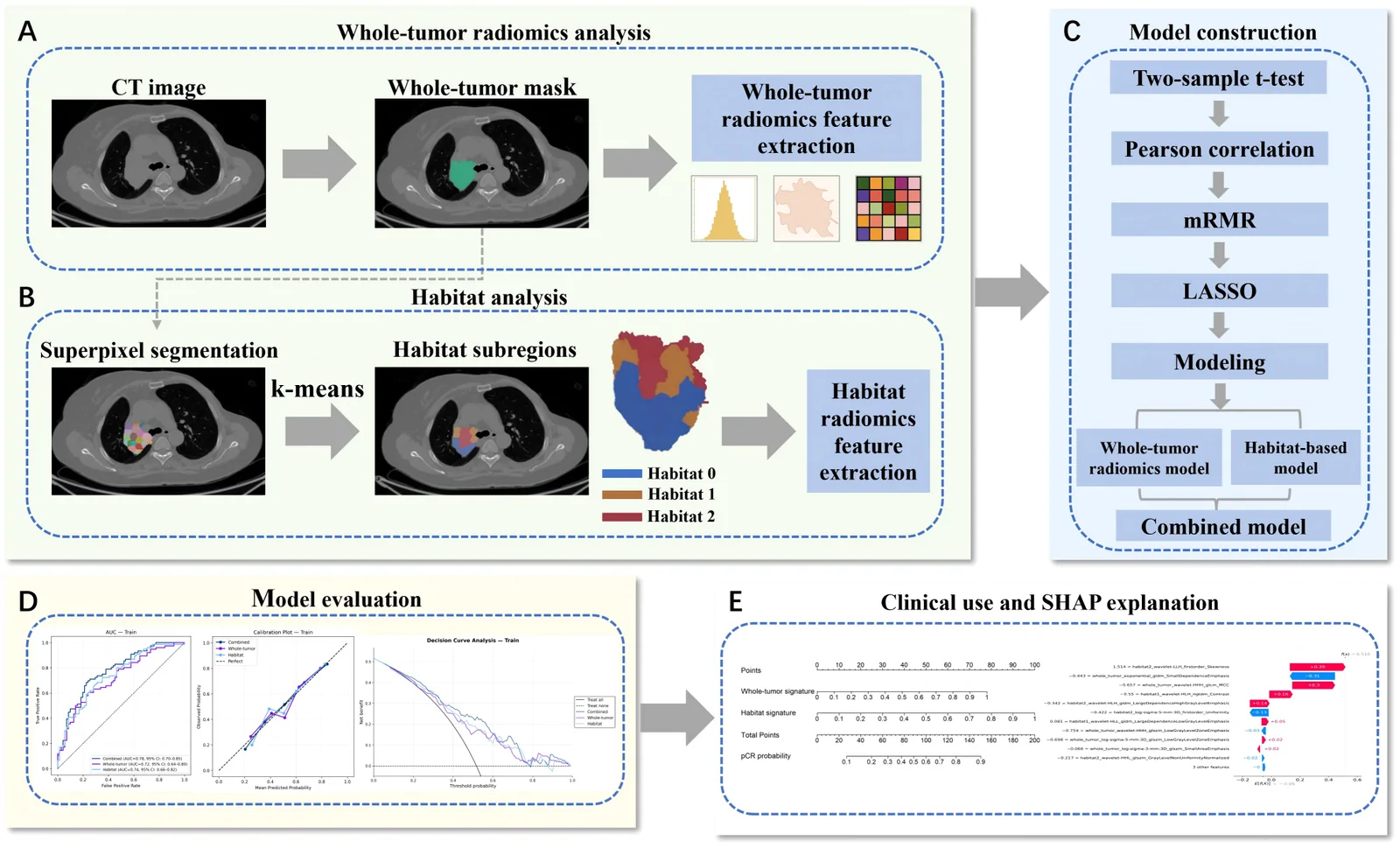
Overall workflow of the study. **(A)** whole-tumor radiomics analysis; **(B)** habitat analysis; **(C)** model construction; **(D)** model evaluation; **(E)** clinical use and SHAP explanation. mRMR, minimum redundancy maximum relevance; LASSO, least absolute shrinkage and selection operator; SHAP, Shapley Additive exPlanations.

### Clinical and pathological data

The clinical and pathological data, including gender, age, smoking history, histopathological type, and clinical stage were all obtained from the electronic medical records. Staging was performed using the 8th edition TNM staging system of the International Association for the Study of Lung Cancer (IASLC). pCR was assessed according to IASLC guidelines. Two experienced pathologists independently evaluated the resected primary tumor and lymph nodes after NACI. Any discrepancies between the two assessments were resolved through joint review and consensus. pCR was defined as the absence of any residual viable tumor cells in both the tumor bed and lymph nodes, corresponding to ypT0 and ypN0.

### Neoadjuvant administration

Patients enrolled in this study received a standardized neoadjuvant regimen comprising platinum-based doublet chemotherapy in combination with immune checkpoint inhibitors (ICIs). The chemotherapeutic agents included cisplatin, lobaplatin, nedaplatin, or carboplatin. For immunotherapy, the majority of patients received PD-(L)1 axis inhibitors, including nivolumab, pembrolizumab, sintilimab, camrelizumab, tislelizumab, envafolimab, SHR-1701, durvalumab, penpulimab, adebrelimab, and toripalimab. A minority of patients received non-PD-(L)1-based immunomodulatory agents specifically, ipilimumab, and/or IBI110.

### Data preprocessing and segmentation

Pixel intensities were clipped to a range of −1000 to 2000 HU to reduce the influence of extreme values and outliers. To standardize voxel spacing, images were resampled to a voxel size of 1 × 1 × 1 mm using linear interpolation. For intratumoral annotation, all DICOM images were uploaded to the Deepwise Multimodal Research Platform (version 2.5.2; https://keyan.deepwise.com). Initial tumor segmentations were automatically generated by the platform. Two radiologists with 5 and 10 years of experience then independently reviewed and manually corrected the automatically generated segmentations. Both radiologists were blinded to the pathological outcomes and to each other’s segmentation results. Radiomics features were extracted separately from both sets of corrected segmentations. Inter-observer reproducibility was evaluated using the intraclass correlation coefficient (ICC) calculated between the two feature sets, and only features with ICC > 0.75 were considered robust and retained for further analysis. The segmentations corrected by the senior radiologist (10 years of experience) were subsequently used as the reference standard for all subsequent feature extraction and model development. **Supplementary Table 1** provides comprehensive details regarding the acquisition and reconstruction parameters of CT scans conducted at two centers.

### Habitat analysis

For each patient, habitat regions were generated within the intratumoral region using a texture-driven clustering framework based on entropy and energy features. Prior to feature extraction, CT images were intensity-normalized using z-score normalization calculated from voxels within the intratumoral region to reduce inter-case intensity variation while preserving intratumoral contrast (26). To improve spatial coherence and reduce voxel-level noise, a spacing-aware Simple Linear Iterative Clustering (SLIC) algorithm was applied to the normalized CT volume, with a target of 100 superpixels and a compactness parameter of 10 (27). The tumor mask was dilated by two iterations to improve boundary stability during SLIC; this dilation was used only for superpixel generation, while subsequent feature calculation and habitat assignment were restricted to the original tumor region. Local entropy and energy were calculated using a 3 × 3 × 3 voxel neighborhood, with entropy calculated using 16 intensity bins and energy defined as the mean squared normalized CT intensity within the local neighborhood. Mean entropy and energy were then calculated for each intratumoral superpixel and standardized using patient-wise z-score normalization before clustering. Habitat partitioning was subsequently performed using K-means clustering (28). Based on prior habitat imaging studies and to mitigate overparameterization, the number of clusters was fixed at three (29, 30). This choice was further evaluated through a sensitivity analysis of K = 2–5 in the training cohort, considering both clustering quality and habitat fragmentation, as detailed in the **Supplementary Methods 1**. To provide consistent relative habitat labeling across patients, the resulting cluster labels were reordered according to their original mean entropy values, with low- (Habitat 0), intermediate- (Habitat 1), and high-entropy regions (Habitat 2) representing relatively low, intermediate, and high intratumoral heterogeneity, respectively.

### Feature extraction

Radiomics features were extracted from the whole-tumor region and each of the three derived habitat regions using the PyRadiomics library (version 3.0.1). A fixed bin width of 25 was used for gray-level discretization. Features were calculated from the original images and multiple filtered image representations, including wavelet decomposition, Laplacian of Gaussian (LoG) filtering with σ values of 3 and 5 mm, and square, square-root, logarithmic, exponential, and gradient transformations. All available PyRadiomics feature classes were enabled, including first-order intensity statistics, three-dimensional shape descriptors, gray-level co-occurrence matrix (GLCM), gray-level run-length matrix (GLRLM), gray-level size-zone matrix (GLSZM), gray-level dependence matrix (GLDM), and neighboring gray-tone difference matrix (NGTDM) features. Parameters not explicitly specified in the extraction script were retained at the default settings of PyRadiomics 3.0.1. Collectively, these features provided a comprehensive quantitative representation of whole-tumor and habitat regions in terms of intensity, geometry, and textural heterogeneity across different spatial frequencies.

### Model construction

Feature selection was performed on the training cohort following a multi-step dimensionality reduction strategy to mitigate redundancy and overfitting. All features were first standardized using z-score normalization based on parameters derived from the training cohort. For each feature, the mean and standard deviation were calculated exclusively from the training cohort, and these training-derived normalization parameters were subsequently applied unchanged to the internal and external validation cohorts. After normalization, univariate filtering was conducted using a two-sample t-test to identify features significantly associated with the outcome (p < 0.05). Subsequently, highly correlated features were removed through pairwise Pearson correlation analysis, using a correlation coefficient threshold of 0.90. The remaining features were then ranked using the minimum redundancy maximum relevance algorithm (31), and the top 20 ranked features were retained for further selection. Next, least absolute shrinkage and selection operator regression with ten-fold cross-validation was applied to identify features with non-zero coefficients (32). After that, logistic regression models were constructed to develop whole-tumor and habitat-based models, each using its respective selected feature set. The combined model was then built using logistic regression based on the features selected from both models. Given the balanced distribution of pCR and non-pCR cases in the training cohort, no class weighting or oversampling was applied during model development. All steps used to define the combined model, including feature standardization, feature selection, and model fitting, were performed exclusively in the training cohort. The resulting model specification was then fixed and applied without modification to the internal and external validation cohorts.

### Statistical analysis

Statistical analysis was performed using R (version 4.4.2, https://www.r-project.org/) and Python (version 3.11.7, https://www.python.org/). Continuous variables were summarized as mean ± standard deviation and compared using the independent samples t-test, whereas categorical variables were expressed as counts and percentages and compared using the chi-square test or Fisher’s exact test, as appropriate. Habitat identification and radiomics feature extraction were independently repeated based on the two sets of tumor segmentations generated by the two radiologists. The reproducibility of the extracted features was evaluated using intraclass correlation coefficients (ICCs), with an ICC > 0.75 considered indicative of good reproducibility. Model performance was evaluated in the training, internal validation, and external test cohorts to assess discrimination, calibration, and clinical utility. Discriminative performance was quantified using the area under the receiver operating characteristic curve (AUC) with corresponding 95% confidence intervals, along with accuracy, sensitivity, specificity, positive predictive value (PPV), and negative predictive value (NPV), and performance differences between models were assessed using the DeLong test. Optimal cutoff values were determined using the Youden index in the training cohort and subsequently applied unchanged to the internal and external validation cohorts to dichotomize predicted probabilities for metric calculation. Model calibration was assessed using calibration intercept, calibration slope, and Brier score. The Hosmer-Lemeshow goodness-of-fit test was applied to evaluate agreement between predicted and observed outcomes. To facilitate clinical interpretation and individualized risk estimation, a nomogram was constructed to visualize the combined model. Clinical usefulness was evaluated using decision curve analysis (DCA) by estimating the net benefit across a range of threshold probabilities. Model interpretability was further assessed using Shapley Additive exPlanations (SHAP) analysis, which quantified and visualized the contribution of individual radiomics features to model predictions. All statistical tests were two-sided, and a p value < 0.05 was considered statistically significant.

## Results

### Patient characteristics

A total of 287 patients who met the inclusion and exclusion criteria and received NACI followed by surgery were included in this study. Among the overall cohort, 135 patients achieved pCR, corresponding to a pCR rate of 47.0%. Most patients were male (84.3%, 242/287). Squamous cell carcinoma was the predominant histological subtype, accounting for 70.0% (201/287) of cases. Patients with clinical stage III disease represented 63.8% (183/287) of the cohort, and the majority received anti-PD-(L)1 therapy (93.7%, 269/287). The study population was divided into a training cohort (n = 147), an internal validation cohort (n = 63), and an external test cohort (n = 77). Across the three cohorts, no statistical differences were observed between the pCR and non-pCR cohorts for most clinical variables, including age, smoking history, TNM stage, histological subtype, or type of immune checkpoint inhibitor therapy. Detailed clinical and pathological characteristics of patients in the training, internal validation, and external test cohorts are summarized in **Table 1**.

**Table 1:** Clinical and pathological characteristics of patients in different cohorts.

Characteristics	Training cohort (n=147)			Internal validation cohort (n=63)			External test cohort (n=77)			
	non-pCR (n=71)	pCR (n=76)	*P*	non-pCR (n=30)	pCR (n=33)	*P*	non-pCR (n=51)	pCR (n=26)	*P*	*P* (among cohorts)
Age(years)	59.9 ± 6.8	61.7 ± 6.3	0.131	61.6 ± 6.5	62.3 ± 6.4	0.644	59.6 ± 9.8	64.0 ± 7.9	0.060	0.506
Gender			0.046			1.000			0.199	0.915
Male	55 (77.5%)	69 (90.8%)		26 (86.7%)	28 (84.8%)		40 (78.4%)	24 (92.3%)		
Female	16 (22.5%)	7 (9.2%)		4 (13.3%)	5 (15.2%)		11 (21.6%)	2 (7.7%)		
Smoking			0.401			0.276			0.523	<0.001
No	15 (21.1%)	11 (14.5%)		10 (33.3%)	6 (18.2%)		23 (45.1%)	9 (34.6%)		
Yes	56 (78.9%)	65 (85.5%)		20 (66.7%)	27 (81.8%)		28 (54.9%)	17 (65.4%)		
TNM stage			0.472			0.579			0.383	<0.001
Stage II	36 (50.7%)	33 (43.4%)		8 (26.7%)	12 (36.4%)		8 (15.7%)	7 (26.9%)		
Stage III	35 (49.3%)	43 (56.6%)		22 (73.3%)	21 (63.6%)		43 (84.3%)	19 (73.1%)		
Histology			0.061			0.094			0.201	0.341
LUAD	19 (26.8%)	11 (14.5%)		11 (36.7%)	6 (18.2%)		20 (39.2%)	5 (19.2%)		
SCC	47 (66.2%)	63 (82.9%)		16 (53.3%)	26 (78.8%)		29 (56.9%)	20 (76.9%)		
Others	5 (7.0%)	2 (2.6%)		3 (10.0%)	1 (3.0%)		2 (3.9%)	1 (3.8%)		
ICIs			0.678			1.000			1.000	0.020
Non-PD-(L)1	8 (11.3%)	6 (7.9%)		2 (6.7%)	2 (6.1%)		0 (0.0%)	0 (0.0%)		
Anti-PD-(L)1	63 (88.7%)	70 (92.1%)		28 (93.3%)	31 (93.9%)		51 (100.0%)	26 (100.0%)		

pCR, pathological complete response; TNM, tumor node metastasis; PD-1,programmed cell death protein 1; PD-L1, programmed death ligand 1; LUAD, lung adenocarcinoma; SCC,squamous cell carcinoma; ICIs, immune checkpoint inhibitors.

P values within each cohort compare non-pCR versus pCR. The P (among cohorts) column compares characteristics among the training, internal validation, and external test cohorts.

### Radiomics features selected in models

Based on pretreatment CT images, 1,502 radiomics features were independently extracted from the whole-tumor region and from each of the three habitat subregions. Among the whole-tumor features, 90.68% (1,362/1,502) had ICC values > 0.75. Similarly, across the three habitat subregions, 82.3% (3,708/4,506) of the extracted radiomics features had ICC values > 0.75. After feature selection, six whole-tumor radiomic features and eight habitat-based radiomic features were retained. These features were used to construct the whole-tumor and habitat-based radiomics models separately. The combined model was composed of radiomic features derived from whole-tumor and habitat subregions. The specific features included in each model are summarized in **Supplementary Table 2**. Detailed feature selection process and final model equation are described in the **Supplementary Methods 2**. The nomogram was used to perform a personalized assessment of the probability of pCR (**Supplementary Figure 1**).

### Model evaluation

The ROC curves of the three prediction models in the training cohort, internal validation cohort, and external test cohort are illustrated in **Figure 3**. Compared with the whole-tumor radiomics model, the habitat-based model showed higher AUCs for pCR prediction across all three cohorts. The largest difference in AUC between the two models was observed in the internal validation cohort, where the habitat-based model achieved an AUC of 0.71 (95% CI, 0.58–0.83) and the whole-tumor radiomics model yielded an AUC of 0.68 (95% CI, 0.54–0.81). The combined model showed performance similar to that of the habitat-based model across the three datasets, with AUCs of 0.78 vs 0.74, 0.72 vs 0.71, and 0.71 vs 0.67 for the combined and habitat-based models, respectively. The DeLong test showed no statistically significant difference between the habitat-based and combined models (p = 0.65), the habitat-based and whole-tumor models (p = 0.77), or the combined and whole-tumor models (p = 0.30) in the external cohort (**Supplementary Table 3**). In terms of sensitivity, the habitat-based and combined models demonstrated generally higher values than the whole-tumor radiomics model in the training and internal validation cohorts (0.66 and 0.68 vs 0.61; 0.67 and 0.67 vs 0.64, respectively). In the external cohort, the habitat-based model maintained a sensitivity of 0.54, whereas the combined model dropped to 0.19, which was only marginally higher than the whole-tumor model’s 0.15. In terms of specificity, the whole-tumor radiomics model showed similar or higher values compared with the habitat-based and combined models across the three cohorts, with the largest difference observed in the external cohort (0.92 vs 0.76 and 0.98). The detailed results are presented in **Table 2**.

**Figure 3:**
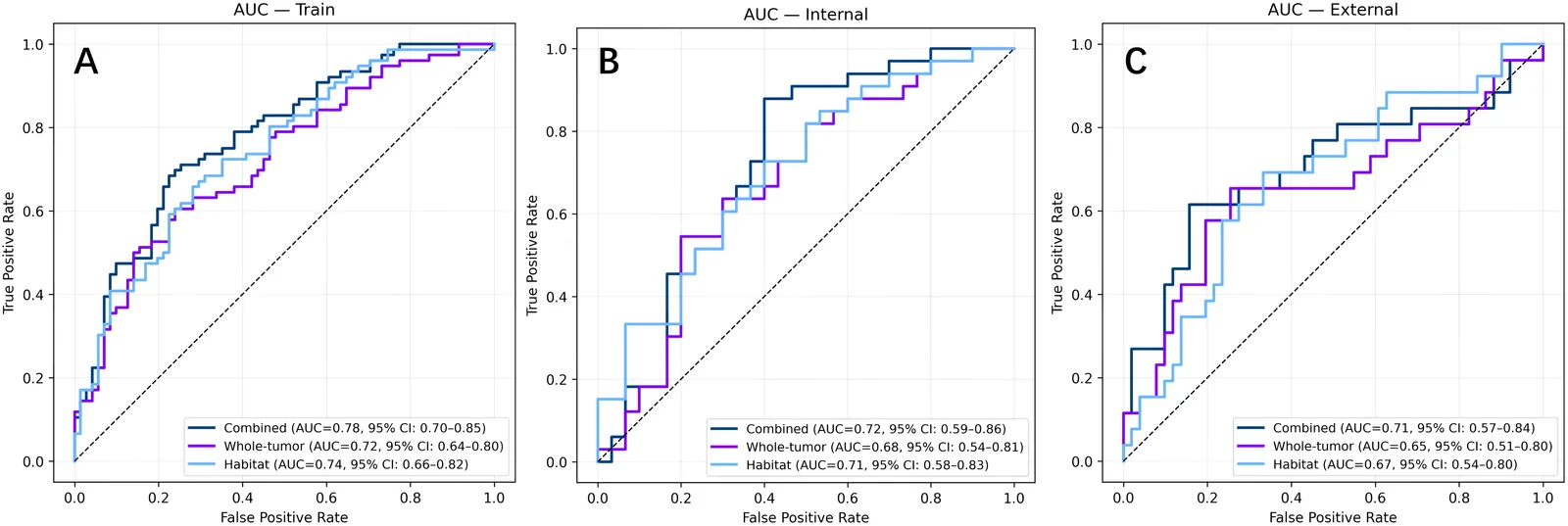
Receiver operating characteristic (ROC) curves of the whole-tumor radiomics, habitat-based, and combined models in the training cohort **(A)**, the internal validation cohort **(B)**, and the external test cohort **(C)**.

**Table 2:** Comparison of performance metrics of different models for predicting pathological complete response.

Cohort	Model	Cut-off	AUC (95%CI)	ACC (95%CI)	Sensitivity (95%CI)	Specificity (95%CI)	PPV (95%CI)	NPV (95%CI)
Training cohort	habitat	0.519	0.74(0.66-0.82)	0.69(0.61-0.76)	0.66(0.54-0.76)	0.72(0.60-0.82)	0.71(0.59-0.82)	0.66(0.55-0.77)
	whole-tumor	0.547	0.72(0.64-0.80)	0.68(0.60-0.75)	0.61(0.49-0.72)	0.76(0.64-0.85)	0.73(0.60-0.83)	0.64(0.53-0.74)
	combined	0.555	0.78(0.70-0.85)	0.73(0.65-0.80)	0.68(0.57-0.79)	0.77(0.66-0.87)	0.76(0.65-0.86)	0.70(0.58-0.79)
Internal validation cohort	habitat	0.519	0.71(0.58-0.83)	0.63(0.50-0.75)	0.67(0.48-0.82)	0.60(0.41-0.77)	0.65(0.46-0.80)	0.62(0.42-0.79)
	whole-tumor	0.547	0.68(0.54-0.81)	0.65(0.52-0.77)	0.64(0.45-0.80)	0.67(0.47-0.83)	0.68(0.49-0.83)	0.62(0.44-0.79)
	combined	0.555	0.72(0.59-0.86)	0.65(0.52-0.77)	0.67(0.48-0.82)	0.63(0.44-0.80)	0.67(0.48-0.82)	0.63(0.44-0.80)
External test cohort	habitat	0.519	0.67(0.54-0.80)	0.69(0.57-0.79)	0.54(0.33-0.73)	0.76(0.63-0.87)	0.54(0.33-0.73)	0.76(0.63-0.87)
	whole-tumor	0.547	0.65(0.51-0.80)	0.66(0.55-0.77)	0.15(0.04-0.35)	0.92(0.81-0.98)	0.50(0.16-0.84)	0.68(0.56-0.79)
	combined	0.555	0.71(0.57-0.84)	0.71(0.60-0.81)	0.19(0.07-0.39)	0.98(0.90-1.00)	0.83(0.36-1.00)	0.70(0.58-0.81)

AUC, area under the curve; CI, confidence interval; ACC, Accuracy; PPV, positive predictive value; NPV, negative predictive value.

DCA of the three models is shown in **Figure 4**. In the training cohort, three models provided positive net benefit over a wide range of threshold probabilities. In the internal validation cohort, the combined model showed clinical net benefit between threshold probabilities of approximately 0.20-0.70, while the habitat-based model provided net benefit primarily at thresholds of approximately 0.30-0.90. In the external test cohort, the combined model maintained positive net benefit across approximately 0.30-0.85, and the habitat-based model across approximately 0.20-0.50. **Figure 5** presents the calibration curves of the models. The Hosmer-Lemeshow test yielded p values greater than 0.05 for both the combined, habitat-based and whole-tumor radiomics models in the training and internal validation cohorts, suggesting no significant difference between the predicted probabilities and the observed pCR outcomes. The calibration intercepts and slopes in these two cohorts were also close to their ideal values of 0 and 1, respectively (**Supplementary Table 4**), indicating acceptable internal calibration performance. However, in the external validation cohort, the calibration performance declined. The calibration slopes were 0.284 for the combined model, 0.461 for the habitat-based model, and 0.500 for the whole-tumor model, indicating that the predicted probabilities were less well calibrated in the external cohort than in the development cohorts.

**Figure 4:**
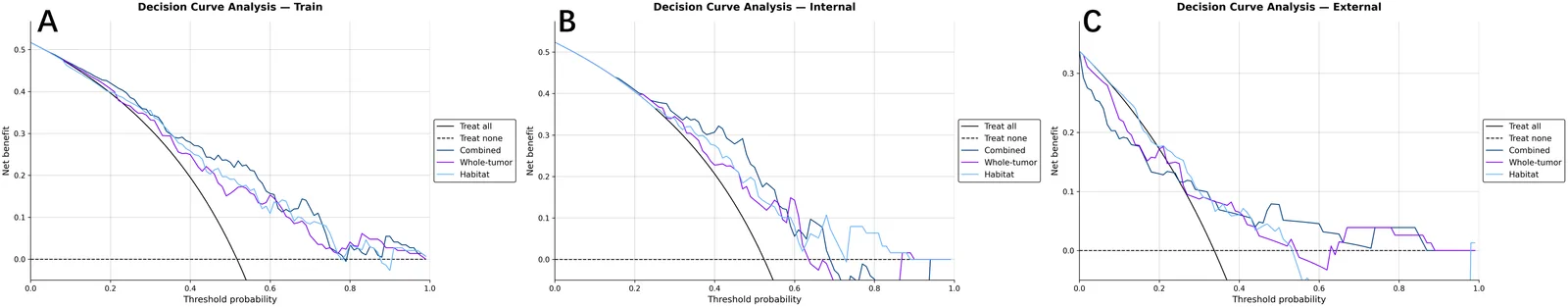
Decision curve analysis (DCA) of the three models in the training cohort **(A)**, internal validation cohort **(B)**, and external test cohort **(C)**.

**Figure 5:**
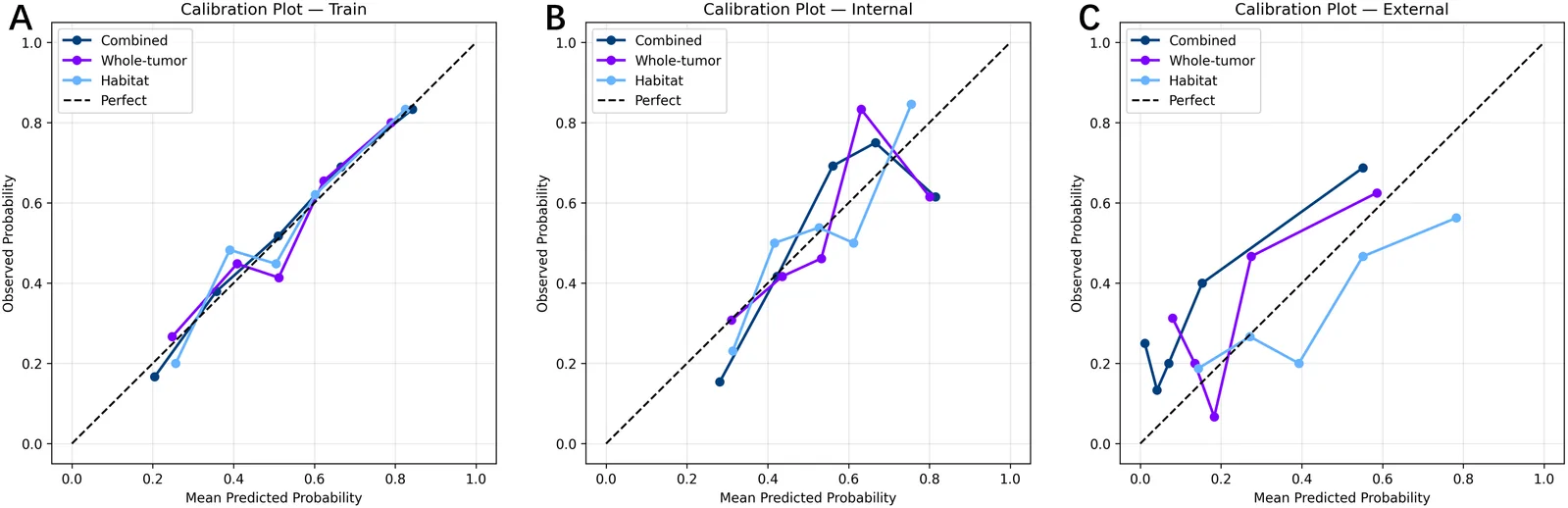
Calibration curves of the predictive models in the training cohort **(A)**, internal validation cohort **(B)**, and external test cohort **(C)**.

### Model interpretability using SHAP analysis

To explore how habitat-related features contributed to model predictions, SHAP analysis was applied to the combined model. The global SHAP contributions of radiomic features from the whole tumor and individual habitat regions are summarized in **Supplementary Figure 2**. The results showed that habitat 2 contributed more than the other individual habitat regions, suggesting that high-entropy regions played an important role in pCR prediction. Two randomly selected examples from the combined model are shown in **Figure 6**. For patient 1, who achieved pCR after NACI, f(x) was 0.516, corresponding to a predicted probability of 0.626 (sigmoid(0.516) = 0.626), which exceeded the 0.555 cutoff, resulting in a correct classification of pCR. The most influential feature was habitat2_wavelet-LLH_firstorder_Skewness. The features describe skewness of the gray-level intensity distribution within habitat 2 and made positive contributions to the model prediction. In contrast, for patient 2, who did not achieve pCR, f(x) was -0.395, corresponding to a predicted probability of 0.402 (sigmoid(-0.395) = 0.402), which fell below the 0.555 cutoff, correctly classifying this patient as non-pCR. In this patient, habitat2_wavelet-LLH_firstorder_Skewness was the main contributing feature and showed negative contributions to the prediction. We hypothesize that habitat2 potentially encompasses necrotic-like low-density components and large solid high-density tumor structures, potentially indicating a resistant and heterogeneous tumor microenvironment associated with poor response.

**Figure 6:**
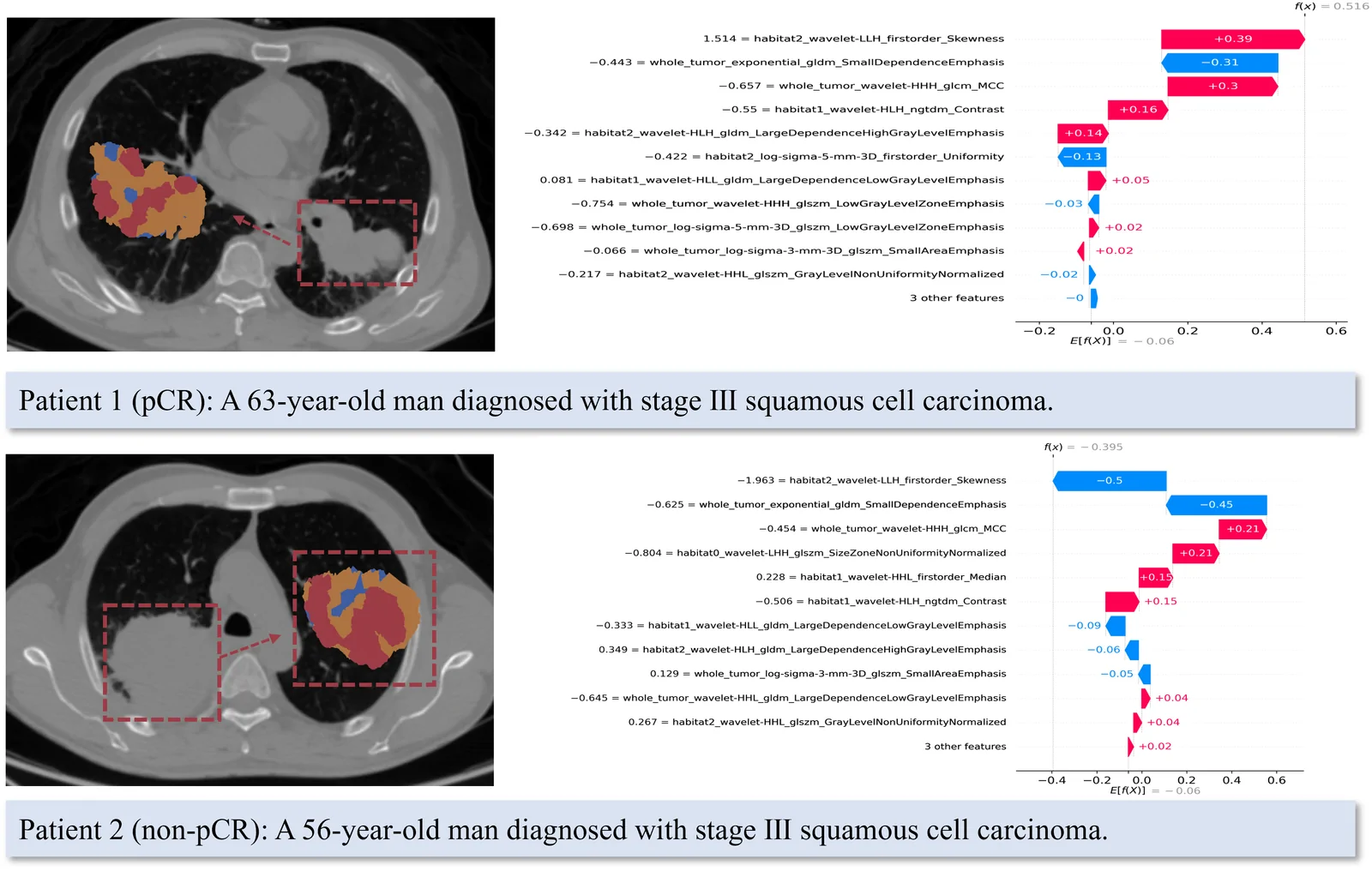
Two randomly selected examples. **(A)** Patient 1 with pCR. The model output f(x) was 0.516 on the log-odds scale, corresponding to a predicted probability of 0.626 (sigmoid(0.516) = 0.626), which exceeded the classification threshold of 0.555, correctly predicting pCR. **(B)** Patient 2 with non-pCR. The model output f(x) was −0.395 (log-odds), corresponding to a predicted probability of 0.402 (sigmoid(−0.395) = 0.402), which fell below the 0.555 threshold, correctly predicting non-pCR. E[f(X)]: expected model output across the training cohort. pCR, pathological complete response; SHAP, Shapley Additive exPlanations.

## Discussion

In patients with NSCLC receiving NACI, pCR has emerged as an important prognostic indicator and has been associated with favorable survival outcomes (5, 33). However, reliable preoperative prediction of pCR remains challenging in clinical practice. In this study, radiomics features derived from pretreatment CT images were analyzed at both the whole-tumor and habitat levels to develop models for pCR prediction. The habitat-based model achieved AUCs of 0.71 and 0.67 in the internal validation and external test cohorts, respectively, and showed higher AUCs than the whole-tumor radiomics model. Although the improvements in AUC did not reach statistical significance in the DeLong test. Moreover, habitat 2 contributed more than the other individual habitat regions, suggesting that these high-entropy characteristics may serve as potential imaging biomarkers for pCR prediction. Such features may provide additional information to support treatment planning and disease management in patients with NSCLC. Building upon these findings, the model may serve as a preoperative risk stratification tool. Patients predicted to have a high probability of achieving pCR may be more likely to achieve a favorable pathological response to the planned NACI, potentially facilitating subsequent minimally invasive or lung-sparing surgical approaches after tumor downstaging. Conversely, identification of patients with a low predicted probability of pCR may prompt closer response monitoring and multidisciplinary evaluation of individualized treatment strategies. Rather than directly determining treatment decisions, the model could provide complementary information for pretreatment risk stratification and clinical decision-making. This approach may help shift the assessment of treatment response from postoperative pathological evaluation toward pretreatment prediction, thereby facilitating more timely and individualized patient management. However, these potential applications remain preliminary and warrant further validation in larger multi-institutional cohorts to support broader generalizability and clinical applicability.

Recent studies have attempted to utilize artificial intelligence techniques for the noninvasive prediction of pCR or MPR. Han et al. analyzed pre-treatment CT images of 243 NSCLC patients undergoing neoadjuvant therapy and developed a nomogram model integrating histology, peritumoral, and habitat radiomics features to predict MPR, achieving an AUC of 0.799 in the test sets (34). The better performance of their model may be due to its integration of multiregional radiomics features and clinical variables, and it is easier to predict MPR (viable tumor cells less than 10%) than pCR (no viable tumor). Another study constructed a radiomics model based on pre-treatment and pre-operative CT, showing that the delta-radiomics model exhibited good performance in predicting pathological response, with AUCs of 0.833,0.716 in two test sets (35). Although integrating multiple time points could yield additional information, our study focused on pre-treatment CT data at a single time point and obtained an AUC of 0.71 in the external test cohort, thereby aligning more closely with early patient stratification. Based on pretreatment non-enhanced and contrast-enhanced CT images, Ye et al. developed three radiomic models, including non-enhanced, contrast-enhanced, and integrated models, to predict pCR in patients with stage IB-III NSCLC undergoing NACI (36). The models achieved AUCs of 0.762, 0.797, and 0.866 in the test cohort, respectively. This performance difference is likely attributable to their incorporation of contrast-enhanced CT features, which provide additional information on tumor vascularity and perfusion characteristics.

Tumor heterogeneity can reflect the differences in genes, phenotypes, functions, and microenvironment within a single neoplastic lesion, which is the basis of tumor progression, therapeutic effect, recurrence, and metastasis (37). Habitat analysis enables quantification of spatial heterogeneity across tumor subregions and has been increasingly applied in lung cancer for pathological subtyping, gene mutation detection, treatment response assessment, and prognostic stratification (29, 30, 38–41). In this study, we investigated the use of a habitat-based model for pCR prediction and found that it showed higher AUCs compared with the whole-tumor model. Furthermore, we integrated the habitat and whole-tumor features to develop a combined model. The combined model did not show significantly improved predictive performance compared to the habitat-based model alone. It may be the reason that 1502 radiomic features were extracted from each habitat subregion, allowing the habitat-based model to capture sufficient predictive information. In contrast, other studies that used fewer habitat features may observe additional benefits from combined modeling (39, 42). Besides, Liu H et al. (43) also found that the habitat model comprehensively captured the core information of tumor heterogeneity, and there might be a high overlap with traditional radiomics in characterizing key tumor biological behaviors (such as proliferation, necrosis, and angiogenesis). They combined a whole-tumor radiomics model with the habitat model, but this integration did not result in a significant performance improvement, which is consistent with our findings.

The association between habitat subregions and pCR was also explored. The features that contributed more were concentrated in habitat 2, which was characterized by high entropy, potentially indicating a complex microenvironment. In the SHAP analysis, features from this subregion mainly contributed to model predictions including habitat2_wavelet-LLH_firstorder_Skewness, which reflected the texture complexity within habitat 2. We hypothesize that irregular texture distribution may stem from the coexistence of multiple components within the tumor, such as cancer cells, necrotic tissue, fibrosis, and inflammatory infiltration, and this may reflect specific biological behaviors, such as high heterogeneity, active cell proliferation, and tumor invasiveness (44–47), leading to adverse pathological response. Furthermore, other studies also reported that a higher degree of heterogeneity in the tumor region correlates with worse immunotherapy outcomes, further supporting our findings (48, 49).

Several limitations of this study should be noted. First, this was a retrospective study, and selection bias cannot be completely excluded. Future large-scale prospective studies are needed to validate these findings. Second, only pretreatment non-contrast chest CT images were used. Contrast-enhanced CT and post-treatment CT were not included, which may have limited the integration of additional imaging features with potential predictive value. However, the use of non-contrast CT is more patient-friendly and avoids the risks associated with contrast agents. It also allows the identification of patients who may benefit from NACI before treatment initiation. Third, tumors were divided into only three habitat subregions. A finer subdivision into more regions may better capture spatial heterogeneity and reveal additional tumor-related changes (50). Fourth, comprehensive immune profiling data (e.g., immune cell infiltration, PD-L1 expression) were not available, and the correlation between radiopathology was not explored. In the future, prospective studies will be conducted to further explore the biological mechanism. Fifth, the reduced calibration slopes observed in the external validation cohort indicate suboptimal calibration and highlight the challenges associated with transporting radiomics-based models across centers. Together with the modest external sensitivities at the prespecified cutoff, these findings suggest that the current models should be regarded as exploratory predictive tools rather than models ready for direct clinical decision-making. Further studies with larger, multi-institutional cohorts are needed to assess model transportability and to investigate recalibration, clinically appropriate threshold selection, and imaging protocol harmonization before potential clinical implementation.

## Conclusions

This study used whole-tumor radiomics and habitat features from pretreatment CT images to develop models for predicting pCR in patients with NSCLC receiving NACI. The habitat-based model showed potential performance, with features from the high-entropy region showing relatively greater contributions among the habitat subregions. The preliminary results suggest that habitat-based features may provide complementary imaging information to support preoperative prediction of treatment response.

## Data Availability

The original contributions presented in the study are included in the article/**Supplementary Material**. Further inquiries can be directed to the corresponding authors.
