# Supplementary material for: Pretreatment CT radiomics for prediction of pathological complete response after neoadjuvant chemoimmunotherapy in NSCLC: a multicenter study incorporating habitat analysis
Source: Front Immunol. 2026 Sep 23;17:1933861. doi: 10.3389/fimmu.2026.1933861 (PMC13642639; doi:10.3389/fimmu.2026.1933861)
Supplement: Supplementary file 1 [file Table1.docx]

**Supplementary Methods**

**1.Habitat region delineation and sensitivity analysis**

To assess the robustness of the predefined three-habitat configuration, a sensitivity analysis was performed in the training cohort for K = 2-5. For each K, clustering quality was evaluated using the Calinski-Harabasz (CH) index, with higher values indicating greater between-cluster separation relative to within-cluster dispersion. In addition, the relative volume of each resulting habitat was calculated to assess progressive habitat fragmentation with increasing K. The number of habitats was determined by considering both cluster separation and the extent of subdivision into small habitats. Based on this trade-off, K = 3 was retained for the primary analysis and subsequently fixed for the internal validation and external test cohorts.

Specifically, sensitivity analysis showed that the mean CH index increased from 178.9 at K = 2 to 240.9, 287.7, and 302.8 at K = 3, 4, and 5, respectively. However, increasing K resulted in progressively smaller habitats: the median proportion of the smallest habitat decreased from 22.7% to 9.1%, 5.4%, and 4.1%, respectively, while the proportion of patients with at least one habitat occupying <5% of the tumor increased from 2.8% to 23.4%, 44.1%, and 61.4%. These findings supported K = 3 as a balance between cluster separation and limiting excessive habitat fragmentation.

**2.Feature selection process and final model equation**

For the whole-tumor radiomics model, 1,362 reproducible features were initially available after ICC filtering. Univariate filtering using a two-sample t-test (p < 0.05) retained 84 features, followed by Pearson correlation filtering (|r| > 0.90), which reduced the number to 41. The remaining features were ranked using the mRMR algorithm. Subsequent . LASSO regression with ten-fold cross-validation resulted in six features for the whole-tumor model. For the habitat-based model, 3,708 reproducible features were initially available after ICC filtering. The t-test retained 118 features, Pearson correlation filtering reduced this number to 64, and the features were retained after mRMR ranking. LASSO regression with ten-fold cross-validation subsequently selected eight features with non-zero coefficients for the habitat-based model. All data-driven feature-selection procedures were performed within the cross-validation framework using the training cohort to minimize information leakage.

The final combined model using the raw features was defined as:

logit(p) = 0.1124930893 + 0.1063411197 * ((habitat2_wavelet-HHL_glszm_GrayLevelNonUniformityNormalized - 0.1888156994) / 0.0611243205) - 0.2844100126 * ((habitat1_wavelet-HLH_ngtdm_Contrast - 0.0086646003) / 0.0112021410) - 0.2121634173 * ((habitat0_wavelet-LHH_glszm_SizeZoneNonUniformityNormalized - 0.3625493726) / 0.0761124556) - 0.3491597440 * ((whole_tumor_log-sigma-3-mm-3D_glszm_SmallAreaEmphasis - 0.5019237074) / 0.0361446779) + 0.3217793724 * ((habitat1_wavelet-HLL_gldm_LargeDependenceLowGrayLevelEmphasis - 0.5618463228) / 0.4228877159) + 0.4641869395 * ((habitat1_wavelet-HHL_firstorder_Median - -0.1839531346) / 0.6728666676) + 0.2557756319 * ((habitat2_wavelet-LLH_firstorder_Skewness - -1.0546888475) / 1.1605073946) - 0.0649265033 * ((whole_tumor_wavelet-HHL_gldm_LargeDependenceLowGrayLevelEmphasis - 5.3444294262) / 4.3792466420) + 0.2931131248 * ((habitat2_log-sigma-5-mm-3D_firstorder_Uniformity - 0.0931604563) / 0.0241053609) + 0.0411569703 * ((whole_tumor_wavelet-HHH_glszm_LowGrayLevelZoneEmphasis - 0.3046324712) / 0.1806244077) + 0.7373750209 * ((whole_tumor_exponential_gldm_SmallDependenceEmphasis - 0.0023893893) / 0.0007790498) - 0.2820022350 * ((habitat2_wavelet-HLH_gldm_LargeDependenceHighGrayLevelEmphasis - 3780.7212065848) / 3735.4083000312) - 0.4152682354 * ((whole_tumor_wavelet-HHH_glcm_MCC - 0.1240980370) / 0.0868559096) - 0.0375546108 * ((whole_tumor_log-sigma-5-mm-3D_glszm_LowGrayLevelZoneEmphasis - 0.0148669099) / 0.0080670927)

**Supplementary Tables**

**Table S1. CT scanning parameters among two centers**

| Parameters | TMUCIH | GPPH |
| --- | --- | --- |
| Manufacturer | Siemens / GE / Philips | Siemens / GE / Philips |
| Tube voltage | 100-120 kVp | 120-140 kVp |
| Tube current | automatically | automatically |
| Reconstruction kernel | soft / hard | soft / hard |
| Matrix | 512 × 512 | 512 × 512 |
| Slice thickness | 1.0 / 1.5 / 1.25 mm | 1.0 / 1.25mm |

**Table S2. Radiomics features used for model construction.**

| **Model** | **Region** | **Radiomics features** |
| --- | --- | --- |
| Whole-tumor radiomics model | Whole tumor | whole_tumor_exponential_gldm_SmallDependenceEmphasis |
|  |  | whole_tumor_log-sigma-5-mm-3D_glszm_LowGrayLevelZoneEmphasis |
|  |  | whole_tumor_log-sigma-3-mm-3D_glszm_SmallAreaEmphasis |
|  |  | whole_tumor_wavelet-HHH_glszm_LowGrayLevelZoneEmphasis |
|  |  | whole_tumor_wavelet-HHH_glcm_MCC |
|  |  | whole_tumor_wavelet-HHL_gldm_LargeDependenceLowGrayLevelEmphasis |
| Habitat-based model | Habitat 0 | habitat0_wavelet-LHH_glszm_SizeZoneNonUniformityNormalized |
|  | Habitat 1 | habitat1_wavelet-HLH_ngtdm_Contrast |
|  |  | habitat1_wavelet-HLL_gldm_LargeDependenceLowGrayLevelEmphasis |
|  |  | habitat1_wavelet-HHL_firstorder_Median |
|  | Habitat 2 | habitat2_wavelet-HHL_glszm_GrayLevelNonUniformityNormalized |
|  |  | habitat2_wavelet-LLH_firstorder_Skewness |
|  | Whole tumor / Habitats | habitat2_log-sigma-5-mm-3D_firstorder_Uniformity |
|  |  | habitat2_wavelet-HLH_gldm_LargeDependenceHighGrayLevelEmphasis |
| Combined model |  | whole_tumor_exponential_gldm_SmallDependenceEmphasis |
|  |  | whole_tumor_log-sigma-5-mm-3D_glszm_LowGrayLevelZoneEmphasis |
|  |  | whole_tumor_log-sigma-3-mm-3D_glszm_SmallAreaEmphasis |
|  |  | whole_tumor_wavelet-HHH_glszm_LowGrayLevelZoneEmphasis |
|  |  | whole_tumor_wavelet-HHH_glcm_MCC |
|  |  | whole_tumor_wavelet-HHL_gldm_LargeDependenceLowGrayLevelEmphasis |
|  |  | habitat0_wavelet-LHH_glszm_SizeZoneNonUniformityNormalized |
|  |  | habitat1_wavelet-HLH_ngtdm_Contrast |
|  |  | habitat1_wavelet-HLL_gldm_LargeDependenceLowGrayLevelEmphasis |
|  |  | habitat1_wavelet-HHL_firstorder_Median |
|  |  | habitat2_wavelet-HHL_glszm_GrayLevelNonUniformityNormalized |
|  |  | habitat2_wavelet-LLH_firstorder_Skewness |
|  |  | habitat2_log-sigma-5-mm-3D_firstorder_Uniformity |
|  |  | habitat2_wavelet-HLH_gldm_LargeDependenceHighGrayLevelEmphasis |

**Table S3. Pairwise comparisons of AUCs between models using paired DeLong tests, with AUC differences and 95% confidence intervals. Z, test statistic from the paired DeLong test; P, two-sided p value.**

| **Comparison** | **Training cohort** | **Internal validation cohort** | **External test cohort** |
| --- | --- | --- | --- |
| Combined vs Whole-tumor | 0.06 (0.00–0.11); Z=1.993; P=0.046 | 0.05 (−0.02–0.12); Z=1.342; P=0.179 | 0.05 (−0.04–0.15); Z=1.045; P=0.296 |
| Habitat-based vs Whole-tumor | 0.02 (−0.06–0.10); Z=0.523; P=0.601 | 0.03 (−0.08–0.13); Z=0.547; P=0.585 | 0.02 (−0.11–0.15); Z=0.287; P=0.774 |
| Combined vs Habitat-based | 0.03 (−0.01–0.08); Z=1.513; P=0.130 | 0.02 (−0.05–0.09); Z=0.511; P=0.609 | 0.03 (−0.10–0.17); Z=0.460; P=0.646 |

**Table S4 .The calibration metrics of different models**

| **Cohort** | **Model** | **Calibration_intercept** | **Calibration_slope** | **Brier_score** |
| --- | --- | --- | --- | --- |
| Training cohort | combined | 0.0022 | 1.067 | 0.1913 |
|  | whole-tumor | 0.0017 | 1.0406 | 0.2109 |
|  | habitat | 0.0044 | 1.043 | 0.2052 |
| Internal validation cohort | combined | -0.1093 | 0.9335 | 0.2104 |
|  | whole-tumor | -0.0408 | 0.7205 | 0.2315 |
|  | habitat | -0.0217 | 1.229 | 0.2157 |
| External test cohort | combined | -0.0221 | 0.284 | 0.2192 |
|  | whole-tumor | -0.0681 | 0.4995 | 0.2156 |
|  | habitat | -0.5845 | 0.461 | 0.2232 |

**Supplementary Figures**

**Fig. S1. The nomogram for clinical use.**

**Fig. S2. Region importance plots based on Shapley Additive Explanations (SHAP). (A) Summed mean absolute SHAP values of the selected features across the whole-tumor and habitat regions. (B) Normalized mean absolute SHAP value per selected feature for each region.**

**Executable inference code**

The executable code for the combined model is shown as follows.

import math

\# ============================================================

INTERCEPT = 0.1124930893

CUTOFF = 0.555

MODEL_PARAMS = {

"habitat2_wavelet-HHL_glszm_GrayLevelNonUniformityNormalized": {

"mean": 0.1888156994,

"std": 0.0611243205,

"coef": 0.1063411197,

},

"habitat1_wavelet-HLH_ngtdm_Contrast": {

"mean": 0.0086646003,

"std": 0.0112021410,

"coef": -0.2844100126,

},

"habitat0_wavelet-LHH_glszm_SizeZoneNonUniformityNormalized": {

"mean": 0.3625493726,

"std": 0.0761124556,

"coef": -0.2121634173,

},

"whole_tumor_log-sigma-3-mm-3D_glszm_SmallAreaEmphasis": {

"mean": 0.5019237074,

"std": 0.0361446779,

"coef": -0.3491597440,

},

"habitat1_wavelet-HLL_gldm_LargeDependenceLowGrayLevelEmphasis": {

"mean": 0.5618463228,

"std": 0.4228877159,

"coef": 0.3217793724,

},

"habitat1_wavelet-HHL_firstorder_Median": {

"mean": -0.1839531346,

"std": 0.6728666676,

"coef": 0.4641869395,

},

"habitat2_wavelet-LLH_firstorder_Skewness": {

"mean": -1.0546888475,

"std": 1.1605073946,

"coef": 0.2557756319,

},

"whole_tumor_wavelet-HHL_gldm_LargeDependenceLowGrayLevelEmphasis": {

"mean": 5.3444294262,

"std": 4.3792466420,

"coef": -0.0649265033,

},

"habitat2_log-sigma-5-mm-3D_firstorder_Uniformity": {

"mean": 0.0931604563,

"std": 0.0241053609,

"coef": 0.2931131248,

},

"whole_tumor_wavelet-HHH_glszm_LowGrayLevelZoneEmphasis": {

"mean": 0.3046324712,

"std": 0.1806244077,

"coef": 0.0411569703,

},

"whole_tumor_exponential_gldm_SmallDependenceEmphasis": {

"mean": 0.0023893893,

"std": 0.0007790498,

"coef": 0.7373750209,

},

"habitat2_wavelet-HLH_gldm_LargeDependenceHighGrayLevelEmphasis": {

"mean": 3780.7212065848,

"std": 3735.4083000312,

"coef": -0.2820022350,

},

"whole_tumor_wavelet-HHH_glcm_MCC": {

"mean": 0.1240980370,

"std": 0.0868559096,

"coef": -0.4152682354,

},

"whole_tumor_log-sigma-5-mm-3D_glszm_LowGrayLevelZoneEmphasis": {

"mean": 0.0148669099,

"std": 0.0080670927,

"coef": -0.0375546108,

},

}

def sigmoid(x):

return 1.0 / (1.0 + math.exp(-x))

def predict_combined_model(raw_features, cutoff=CUTOFF, verbose=True):

"""

Parameters

----------

raw_features : dict

Dictionary containing the raw PyRadiomics feature values.

Keys must exactly match MODEL_PARAMS.

cutoff : float

Classification cutoff. Default = 0.555.

Returns

-------

result : dict

logit, probability, predicted_class,

and standardized feature values.

"""

missing = [f for f in MODEL_PARAMS if f not in raw_features]

if missing:

raise ValueError(

"Missing required features:\n" + "\n".join(missing)

)

logit = INTERCEPT

z_scores = {}

contributions = {}

for feature, params in MODEL_PARAMS.items():

x = float(raw_features[feature])

mean = params["mean"]

std = params["std"]

coef = params["coef"]

z = (x - mean) / std

contribution = coef * z

z_scores[feature] = z

contributions[feature] = contribution

logit += contribution

probability = sigmoid(logit)

predicted_class = int(probability >= cutoff)

if verbose:

print("=" * 90)

print("Final combined model prediction")

print("=" * 90)

print(f"Logit	: {logit:.10f}")

print(f"Probability : {probability:.10f}")

print(f"Cutoff	: {cutoff:.3f}")

print(

"Prediction : "

+ ("pCR" if predicted_class == 1 else "non-pCR")

)

print("\nFeature details:")

for feature in MODEL_PARAMS:

print(

f"\n{feature}\n"

f" raw value	= {raw_features[feature]}\n"

f" mean	= {MODEL_PARAMS[feature]['mean']:.10f}\n"

f" std	= {MODEL_PARAMS[feature]['std']:.10f}\n"

f" z-score	= {z_scores[feature]:.10f}\n"

f" coefficient = {MODEL_PARAMS[feature]['coef']:.10f}\n"

f" contribution = {contributions[feature]:.10f}"

)

return {

"logit": logit,

"probability": probability,

"predicted_class": predicted_class,

"z_scores": z_scores,

"contributions": contributions,

}

\# ============================================================

\# Example usage

\# Replace the values below with raw PyRadiomics feature values

\# ============================================================

if __name__ == "__main__":

patient_features = {

"habitat2_wavelet-HHL_glszm_GrayLevelNonUniformityNormalized": 0.20,

"habitat1_wavelet-HLH_ngtdm_Contrast": 0.010,

"habitat0_wavelet-LHH_glszm_SizeZoneNonUniformityNormalized": 0.35,

"whole_tumor_log-sigma-3-mm-3D_glszm_SmallAreaEmphasis": 0.50,

"habitat1_wavelet-HLL_gldm_LargeDependenceLowGrayLevelEmphasis": 0.60,

"habitat1_wavelet-HHL_firstorder_Median": -0.10,

"habitat2_wavelet-LLH_firstorder_Skewness": -1.00,

"whole_tumor_wavelet-HHL_gldm_LargeDependenceLowGrayLevelEmphasis": 5.0,

"habitat2_log-sigma-5-mm-3D_firstorder_Uniformity": 0.10,

"whole_tumor_wavelet-HHH_glszm_LowGrayLevelZoneEmphasis": 0.30,

"whole_tumor_exponential_gldm_SmallDependenceEmphasis": 0.0025,

"habitat2_wavelet-HLH_gldm_LargeDependenceHighGrayLevelEmphasis": 3800.0,

"whole_tumor_wavelet-HHH_glcm_MCC": 0.12,

"whole_tumor_log-sigma-5-mm-3D_glszm_LowGrayLevelZoneEmphasis": 0.015,

}

result = predict_combined_model(patient_features)
